# Optimizing molecular testing of lung cancer needle biopsy specimens: potential solutions from an interdisciplinary qualitative study

**DOI:** 10.1186/s12931-023-02321-9

**Published:** 2023-01-17

**Authors:** Florian J. Fintelmann, Nikki A. Martin, Ismail Tahir, Elissa M. Quinn, Timothy C. Allen, Lija Joseph, Boris Nikolic, Christopher Lee

**Affiliations:** 1 Department of Radiology, Division of Thoracic Imaging and Intervention, Massachusetts General Hospital, 55 Fruit Street, Boston, MA 02114 USA; 2 LUNGevity Foundation, Bethesda, MD USA; 3 Blueprint Medicines, Boston, MA USA; 4 Beaumont Hospital, Royal Oak, MI USA; 5 Lowell General Hospital, Lowell, MA USA; 6 Wyoming Valley Radiology Associates, Wilkes-Barre General Hospital, Wilkes-Barre, PA USA; 7 Department of Imaging, Cedars-Sinai Medical Center, Los Angeles, CA USA

**Keywords:** Lung cancer, Lung needle biopsy, Molecular analysis, Biomarker testing, Genomic analysis

## Abstract

**Background:**

Molecular testing can detect actionable genomic alterations and tumor cell surface proteins in patients with non–small cell lung cancer (NSCLC). However, utilization remains suboptimal, representing missed treatment opportunities. This study aimed to identify challenges and potential solutions to obtaining percutaneous lung needle biopsy specimens for successful molecular testing in patients with advanced NSCLC.

**Methods:**

This interdisciplinary qualitative study included ten radiologists and four pathologists from academic and community settings across the United States who routinely perform and analyze percutaneous lung needle biopsies. Participants underwent semi-structured one-on-one interviews (Phase 1). Interview questionnaires were constructed based on a literature review of key lines of inquiry and conducted by professional market researchers using the theoretical domains framework. Primary barriers to molecular testing were identified using thematic analysis. Subsequently, multidisciplinary focus groups were convened to identify potential solutions (Phase 2).

**Results:**

Four themes emerged as barriers to molecular testing and were matched to the clinical workflow: (1) biopsy request, (2) biopsy procedure, (3) specimen analysis, and (4) communication. The nineteen potential solutions included adding a “checkbox” to indicate molecular testing in the biopsy request, leveraging pre-procedural imaging to guide biopsies, conserving tissue through appropriate allocation strategies and next generation sequencing panels instead of sequential single-gene assays, instituting reflex-molecular testing upon NSCLC diagnosis, tracking and communicating biopsy outcomes at multidisciplinary tumor boards, and improving integration of radiologists and pathologists into oncology care teams.

**Conclusions:**

Potential solutions exist to increase successful molecular testing of lung needle biopsy specimens in patients with advanced NSCLC.

## Introduction

Nearly three in four patients with non–small cell lung cancer (NSCLC) present with advanced disease at the time of diagnosis [1–3]. Sixty to 80% of those patients (Western versus Asian populations, respectively) harbor genomic alterations amenable to targeted therapy [4, 5]. Separately or in conjunction, patients may express tumor cell surface proteins rendering them eligible for immunotherapy [6, 7]. Patients with advanced NSCLC who receive first-line targeted therapy or immunotherapy have improved objective response rates [8, 9], longer progression-free survival, and longer overall survival [10–15]. Molecular testing (also referred to as biomarker testing, genomic analysis, molecular profiling, or molecular analysis) of lung needle biopsy specimens is crucial for identifying subsets of patients who are candidates for targeted therapy or immunotherapy. Despite recommendations by the National Comprehensive Cancer Network (NCCN) for molecular testing in all cases of advanced NSCLC [6], testing levels and results remain suboptimal [2, 16, 17], which translates to missed treatment opportunities [18].

While percutaneous lung needle biopsy is safe in the setting of advanced NSCLC [19, 20], confusion persists among radiologists and pathologists regarding how much tissue is needed [2], as well as the optimal preparation of tissue for histopathology and molecular testing [20, 21]. Insufficient material for molecular testing is procured approximately 40% of the time [8]. While efforts are underway to speed up molecular testing, the need for repeat biopsy can lengthen a patient’s wait to initiate therapy by an average of 57 days (range 31–90 days) [22, 23].

This study sought to identify real-world challenges and potential solutions to obtaining percutaneous lung needle biopsy specimens for successful molecular testing in patients with advanced NSCLC.

## Methods

### Study design

An interdisciplinary qualitative study consisting of semi-structured interviews and focus group discussions with radiologists and pathologists was conducted. Participants voluntarily agreed to have their interviews and focus group discussions transcribed and analyzed for the purposes of this study. The study was exempt from institutional review board approval [45 Code of Federal Regulations 46.104 (d) (2)].

### Participants

**Fig. 1:**
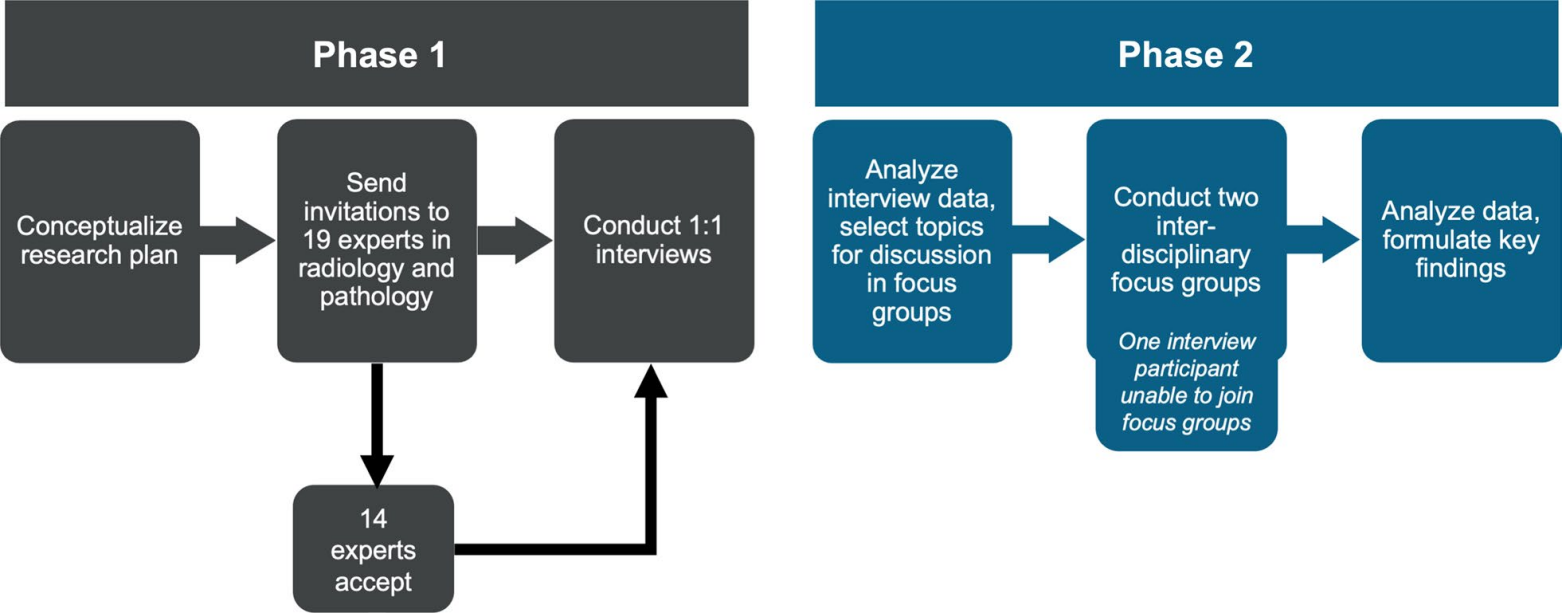
Overview of Study Design and Participants Included

**Fig. 2:**
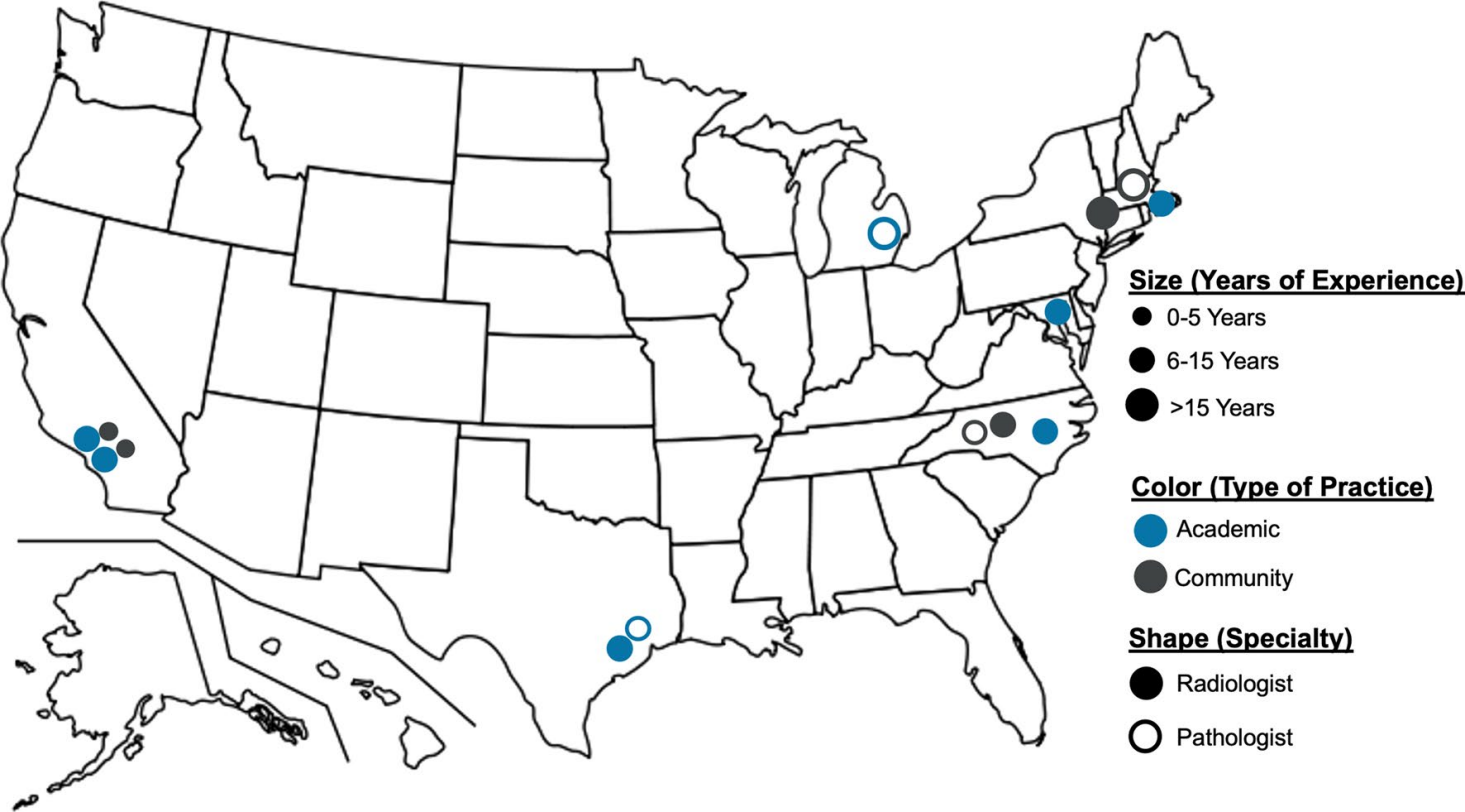
Geographical overview of participants based on years of experience, type of practice, and speciality

The authors identified participants in an iterative process, targeting interventional and thoracic radiologists and pathologists across the United States who routinely perform and analyze percutaneous lung needle biopsies. Participants worked in academic or community-based clinical settings, and four were involved in developing the latest College of American Pathologists (CAP) thoracic biopsy guidelines [24]. Of 11 invited physicians, six agreed to participate. Eight additional radiologists and pathologists from community and academic sites in the southern and southeastern United States were invited to increase geographic and practice setting diversity. Seven out of eight agreed to participate. In total, 19 physicians were invited, and the final panel consisted of ten radiologists (six academic, four community) and four pathologists (two academic, two community). The median number of years in practice was 12 (range 3–32 years) (Figs. 1, 2).

### Data collection

Data collection occurred in two phases. In phase 1, trained moderators from the market research firm (Edge Research, Arlington, VA) conducted 14 semi-structured one-on-one 60-min interviews via videoconference from February to March 2021. Researchers developed lines of inquiry which included the following: goals of lung needle biopsy, awareness of molecular testing, typical lung biopsy scenarios and procedures, perceived and experienced challenges to obtaining tissue samples of sufficient quantity and quality for molecular testing, workflow, and communications between requesting providers and other team members, the role of liquid biopsy, and suggestions for improvement. The theoretical domains framework for the interview focused on knowledge, skills, intentions, context, resources, and consequences [25]. Questions are listed in Table 1. Some lines of questioning were directed only to radiologists; others were specifically directed to pathologists. An independent professional online transcription service recorded and transcribed all interviews.

**Table 1:** Guide for semi-structured one-on-one interviews (phase 1)

Molecular testing knowledge and relationship to lung needle biopsy
1. What have you heard about molecular testing specifically for lung cancer? 2. How (if at all) has molecular testing changed or impacted lung needle biopsy procedures? 3. To what extent are you aware of whether providers are requesting this type of testing? 4. How much do you know about the role that tissue obtained from biopsy plays in the testing process for lung cancer biomarkers? 5. What role does tissue quantity play in molecular testing for non-small cell lung cancer? For example, minimum sample requirements, standards around tissue acquisition for molecular testing? 6. Are there implications for testing based on the quality of the sample? What are those? 7. What does it mean to have a sample that is “representative” of the tumor? 8. How much of a priority is it to have enough tissue sample to be able to test for biomarkers that do not have currently approved treatments? What about biomarkers in clinical trials? 9. What do you know or what have you heard about liquid biopsy (blood-based molecular testing)? Does the promise of liquid biopsy influence how you think about your role? Could it influence the importance of your role for acquiring tissue for molecular testing?
Challenges to quality and quantity of tissue acquisition
1. What do you see as the most significant challenges to getting samples of sufficient amount and quality in a lung needle biopsy? a. What other members of the healthcare team are involved? How do their interactions make this more or less challenging? b. Is this challenge unique to the setting you are in? What could help? c. What guidelines exist to standardize how your profession conducts lung needle biopsy so that patients receive standard of care comprehensive molecular testing? Are these guidelines well utilized/known by other radiologists? 2. Breaking down the challenges step-by-step, does the request for a lung needle biopsy usually come from an oncologist, pulmonologist, or does it depend? a. Do you know that the tissue will be used for molecular testing before the lung needle biopsy procedure begins? b. Are there differences in what is done based on the request and the type of provider writing the request? c. What, if any, challenges to communication are there? d. If the requesting provider is far away or at a different hospital, does that create any challenges? e. Who handles communications to prepare the patient for what they will experience and need to do during the procedure? f. How (if at all) does communication with the patient and patient preparedness impact the success of the procedure? 3. How about challenges with the procedure itself? Are there specific challenges to the quality and quantity of sample based on a. Type of biopsy—core vs FNA b. Type of imaging equipment used c. Tumor location d. Tumor type 4. To what extent is the desire to be less invasive and less stressful for the patient at odds with getting sufficient tissue? 5. What are the challenges with performing multiple passes? a. Are there tradeoffs between doing multiple passes and patient well-being? 6. How do you now if the sample is sufficient and if you have samples that are representative of the tumor? a. When (if ever) is a pathologist on site to evaluate the sample before it is sent for testing? b. How might that be helpful? How does this work? 7. Are there challenges with preparation of tissue for transport or transport itself? a. How does tissue get from the collection site to the molecular pathology lab? Do you play a role in determining how tissue is transported to the lab? b. Are you aware of any potential problems with tissue handling? c. Is dealing with these labs that do molecular testing different than other labs for histologic staining? d. Are there any unique challenges for hospital systems in the transport of samples to the lab that are different from a specialized cancer center or a university/research hospital? 8. Do you get feedback from the lab or pathologist on the quality or quantity of the sample? a. If so, how does it get to you? What is helpful about that? b. If not, what might be helpful about it? 9. Are there aspects of the lung needle biopsy process that are made more difficult because of the insurance or reimbursement policies? For example, challenges with patients on Medicare, Medicaid, or private insurance? a. Are there challenges with reimbursing more than one biopsy due to insufficient tissue for molecular testing?

Semi-structured on-on-one interviews were conducted by market researchers independent of the study. All study participants were interviewed (n = 14). *FNA* fine needle aspiration

In phase 2, two 90-min multidisciplinary focus groups were convened via videoconference in April and May 2021. The first group was comprised of six participants (four radiologists, two pathologists), and the second comprised of seven participants (six radiologists, one pathologist), all of whom had participated in the phase 1 interviews. Discussions were led by a trained moderator from the market research firm who presented findings from the semi-structured interviews and facilitated discussions to elicit divergent and convergent views regarding the interview findings and potential solutions (Table 2). One pathologist was unable to attend the focus groups and instead conducted a one-on-one discussion with the moderator (Table 2).

**Table 2:** Guide for focus group discussion (phase 2)

Pre-procedure and biopsy request – What information do you need to know about the patient ahead of the lung needle biopsy? – What information do you need to know about the lesion? – What is the best way to receive that information? Differences by setting? – How helpful are tumor boards? Who needs to be present?
Biopsy procedure and specimen analysis – What technology is most helpful to guide needle placement? – What are the downsides of each technology? – What are the barriers to getting that technology? Differences by setting? – What is most helpful to assess sample quality? – How much sample do you need for molecular testing? How does tissue quality impact that? – What role does the testing technology play? How can that be improved?
Post-procedure and communication – What is the best way to receive feedback on the sample after molecular testing? – If you do not share an electronic medical record with the pathologist, how could you receive that feedback? – What role does the test report play?

The focus group guide was developed based on the themes extracted from the semi-structured one-on-one interviews (phase 1). Focus group discussions were conducted by independent market researchers. Thirteen participants were present for the focus group (n = 13). One pathologist was unable to attend the focus group and was interviewed using the same guide

### Data analysis

The market researchers analyzed the transcripts of the recorded one-on-one interviews and focus groups to identify patterns for each discussion topic, applying tags to identify responses as a radiologist or pathologist perspective. Interview responses were sorted by question topic and further stratified by sub-topic. Researchers applied a thematic analysis to sort narrative text data into themes based on their relative frequency of appearance [26]. Each topic was examined for convergence or divergence with regard to their importance and impact on molecular testing among the panel. Based on the emerging themes, barriers and solutions were extracted and summarized directly as transcribed from the interviews and focus groups.

## Results

**Fig. 3:**
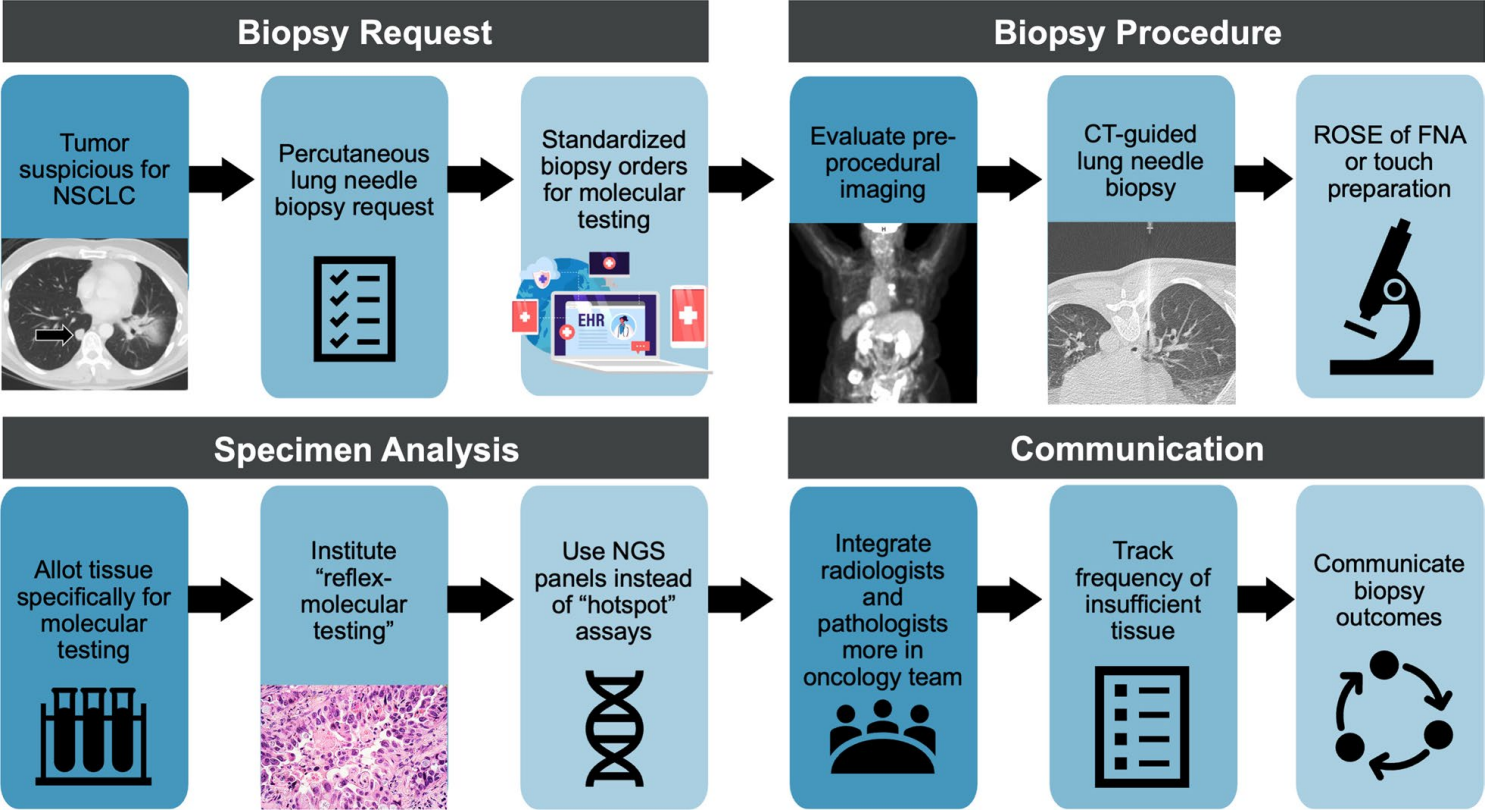
Overview of key barriers and potential solutions to obtaining percutaneous lung needle biopsy specimens for successful molecular testing. *CT* computed tomography, *FNA* fine needle aspiration, *NGS* next generation sequencing, *NSCLC* non-small cell lung cancer, *ROSE* rapid on-site evaluation

Four themes emerged as barriers to obtaining percutaneous lung needle biopsy specimens for successful molecular testing in patients with advanced NSCLC and were matched to the clinical workflow as follows: (1) biopsy request, (2) biopsy procedure, (3) specimen analysis, and (4) communication (Fig. 3). Potential solutions to increase successful molecular testing of lung needle biopsy specimens were also matched to the clinical workflow (Table 3).

**Table 3:** Potential solutions to increase comprehensive molecular testing of lung needle biopsy specimens in patients with advanced non-small cell lung cancer

Biopsy request 1. Educate referring clinicians that NSCLC workup requires comprehensive molecular testing through designated training modules or workshops 2. Add a “checkbox” to the biopsy request in the electronic medical record to indicate the request for molecular testing. Have the requesting provider check this box in all suspected and confirmed cases of advanced NSCLC 3. Pre-screen lung needle biopsy requests and call the referring provider for clarification if the need for molecular testing is unclear
Biopsy procedure 4. Review available imaging prior to the procedure and stratify targets by imaging characteristics, if possible. For example, assess FDG and intravenous contrast to avoid areas of necrosis 5. Use real-time CT guidance during the procedure 6. Obtain multiple tissue cores using coaxial technique while angling the biopsy device in all four quadrants 7. Sample the periphery of large tumors to avoid areas of central necrosis 8. During ROSE, have the cytotechnologist verify that the specimen contains viable tumor cells 9. Collect at least four tissue cores once ROSE confirms viable tumor cells 10. Consider a higher tolerance for potential risk and obtain additional cores if molecular testing is likely to affect survival, especially in suspected advanced NSCLC
Specimen analysis 11. Clarify how to allocate the specimens depending on institutional requirements 12. Use NGS panels instead of sequential single-gene tests to conserve tissue and obtain more comprehensive results 13. If feasible, bank tissue for future molecular testing 14. Institute “reflex-molecular testing” following histologic diagnosis of NSCLC to reduce turnaround time
Communication 15. Track frequency of insufficient tissue, reasons for insufficiency, molecular testing results (especially true negatives versus insufficient sample), complications from the biopsy procedure, and patient treatment outcomes 16. Integrate radiologists who perform lung needle biopsies more closely into the oncology care team and foster a culture of continuous feedback and follow-up 17. Institute a multidisciplinary tumor board with data-driven discussions to increase the quality of lung needle biopsies 18. Report tissue adequacy and cellularity in uniquely identified fields (*not* free-form comments) in the molecular testing report 19. Institute patient navigators to facilitate multidisciplinary follow-up on molecular testing outcomes

Solutions are listed by barriers to the lung needle biopsy workflow. *CT* computed tomography, *FDG* 18-fluorodeoxyglucose, *FNA* fine needle aspiration, *NGS* next generation sequencing, *NSCLC* non-small cell lung cancer, *ROSE* rapid on-site evaluation

### Biopsy request

#### Barriers

Radiologists reported lack of clarity regarding the goal of percutaneous lung needle biopsies. Most radiologists stated that they viewed lung biopsies primarily as a tool to confirm the diagnosis, histology, or staging of lung cancer. Some radiologists noted that the goal of a needle biopsy was to obtain tissue samples sufficient in both quantity and quality, and to avoid repeat biopsies. Although radiologists acknowledged the NCCN recommendations for molecular testing as part of the diagnostic workup of advanced NSCLC, molecular testing was still perceived as a secondary goal. Additionally, interviewees noted that biopsy requests were inconsistent in indicating the need for molecular testing.

#### Potential solutions

- Educate referring clinicians that NSCLC workup requires comprehensive molecular testing through designated training modules or workshops.
- Add a “checkbox” to the biopsy request in the electronic health record to indicate the request for molecular testing. Have the requesting provider check this box in all suspected and confirmed cases of advanced NSCLC.
- Pre-screen lung needle biopsy requests and call the referring provider for clarification if the need for molecular testing is unclear.

### Biopsy procedure

#### Barriers

Participants noted numerous barriers to obtaining tissue that is both high quality and of sufficient quantity: (1) limited pre-procedural imaging due to insurance coverage, (2) radiologist skill and comfort with image-guided lung needle biopsies, (3) lesion size, location, and density, (4) patient tolerance for the procedure, (5) lack of standardization for intraprocedural tissue evaluation, and (6) differing lab instructions for sample preparation. Participants cited being unsure of how much tissue was “enough.”

Participants differed in their opinions regarding the best approach for judging sample adequacy. While rapid on-site evaluation (ROSE) by a pathologist was described as the best practice in theory, radiologists noted that accurate assessment of a biopsy sample with minimal processing during ROSE requires extensive experience, lengthens the needle dwell time, increases the patient’s risk for complications, may not change the procedural steps for the radiologist, and does not appear to affect patient callback rate. Some pathologists noted that ROSE was not always necessary, may not be feasible in all cases, and may be more helpful for inexperienced radiologists. ROSE seemed more prevalent in academic settings due to pathologist availability. Most radiologists did not perform fine needle aspirations (FNA) and performed core biopsies only. Needle size was a matter of personal preference, and there was no consensus that larger needles were better for molecular testing.

#### Potential solutions

- Review available imaging, including computed tomography (CT) and positron emission tomography (PET), prior to the procedure and stratify targets by imaging characteristics. For example, absence of 18-fluorodeoxyglucose uptake and intravenous contrast enhancement can identify necrotic tissue.
- Utilize real-time (i.e., fluoroscopic) CT guidance during the procedure.
- Obtain multiple tissue cores using coaxial technique while angling the biopsy device in all four quadrants
- Target the periphery of large tumors to avoid central necrosis.
- During ROSE, have the cytotechnologist verify that the specimen contains viable tumor cells.
- Collect at least four tissue cores once ROSE confirms viable tumor cells.
- Consider a higher tolerance for potential risk and obtain additional tissue cores if molecular testing is likely to affect survival, especially in suspected advanced NSCLC.

### Specimen analysis

#### Barriers

Participants noted (1) a lack of awareness of current guidelines on molecular testing for patients with advanced NSCLC, (2) varying testing methods by lab, (3) varying sample requirements between multiple available testing platforms, and (4) rapidly changing best practices. Although most study participants were aware of the 2020 CAP thoracic biopsy guidelines, awareness of current NCCN guidelines for molecular testing was low. Some participants mentioned relying on institutional or departmental guidelines.

#### Potential solutions

- Clarify how to allocate the specimens depending on institutional laboratory requirements.
- Perform next generation sequencing (NGS) assays instead of sequential single-gene assays to conserve tissue and provide more comprehensive molecular testing.
- If feasible, bank tissue for future molecular testing.
- Institute “reflex-molecular testing” following histologic diagnosis of NSCLC to reduce turnaround time.

### Communication

#### Barriers

Participants reported the absence of formal, consistent communication feedback loops between radiologists, pathologists, and clinical care teams regarding molecular testing results of lung needle biopsy specimens. Feedback was lacking on amount of viable tumor cells and molecular testing success unless a repeat biopsy was requested. Hospital-based radiologists can access biopsy results through the electronic medical record, while radiologists outside of hospital systems may not have access at all to the results.

#### Potential solutions

**Fig. 4:**
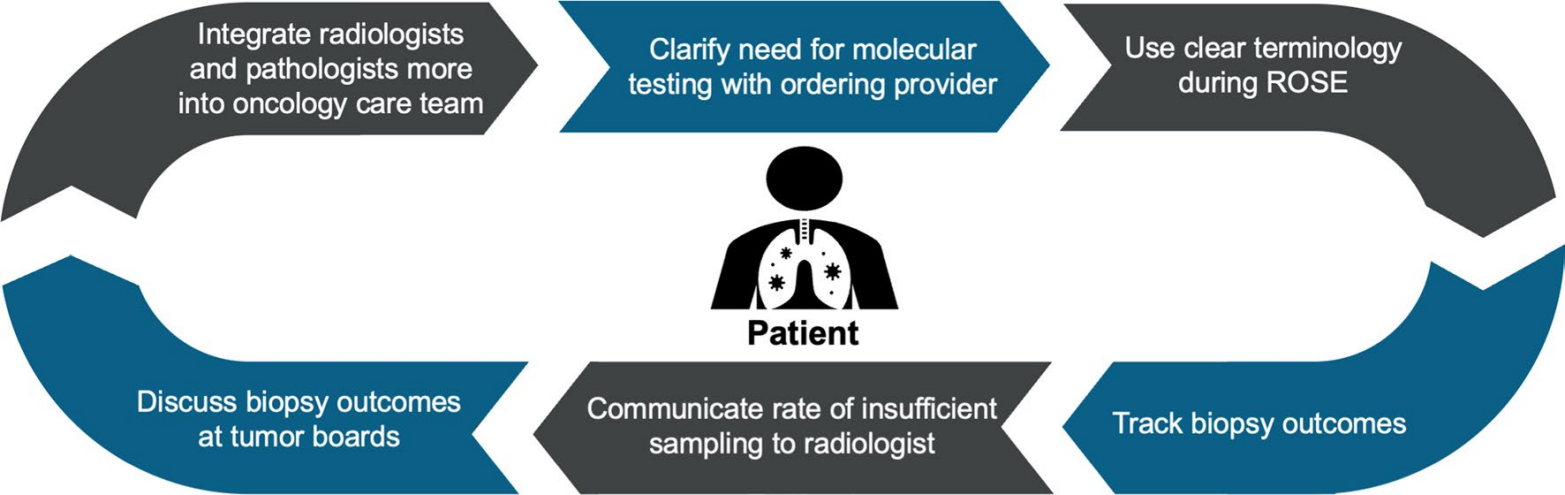
Communication feedback loops to optimize molecular testing. *ROSE* rapid on-site evaluation

- Track frequency of insufficient tissue, reasons for insufficiency, molecular testing results (especially true negatives versus insufficient samples), and complications from the biopsy procedure.
- Integrate radiologists who perform lung needle biopsies more closely into the oncology care team and foster a culture of continuous feedback and follow-up (Fig. 4).
- Include data-driven discussions of biopsy outcomes into multidisciplinary tumor boards.
- Report amount of viable tumor cells in uniquely identified fields (*not* free-form comments) in the molecular testing report [27].
- Institute patient navigators to facilitate follow-up on molecular testing outcomes.

### Liquid biopsy

Panelists agreed that liquid biopsy does not obviate the need for molecular testing of tissue samples and is not currently sensitive enough to replace tissue sampling. However, panelists agreed that liquid biopsy would likely play a role in the future, such as in the setting of treatment failure. The panel was uncertain whether insurance companies would reimburse molecular testing of both tissue and liquid biopsies.

## Discussion

This interdisciplinary qualitative study sought to identify challenges and potential solutions to obtaining percutaneous lung needle biopsy specimens for successful molecular testing in patients with advanced NSCLC. Four themes emerged as barriers and were mapped to the clinical workflow: (1) biopsy request, (2) biopsy procedure, (3) specimen analysis, and (4) communication. Nineteen potential solutions were identified and seem intuitive. This study is important because it identifies barriers and potential solutions to enhance molecular testing in patients with advanced NSCLC, a prerequisite to translate the discovery of novel targeted therapies and immunotherapy into potential cures [15].

Potential solutions identified in this study (Table 3) are in line with prior literature. A prior multidisciplinary panel of pathologists, interventional radiologists, oncologists and radiation oncologists concluded that pathologist-instituted reflex testing upon confirmation of non-squamous NSCLC can improve the timeliness of molecular testing [28]. The panel also highlighted the importance of collaboration and communication between physicians involved in diagnosing patients with NSCLC [28]. Gregg et al. offered similar strategies to improve molecular testing, including ordering molecular tests as soon as advanced NSCLC is suspected, conducting liquid biopsies early in the diagnostic pathway, and instituting pathology-directed reflex testing [29]. A recent report based on data from the Diaceutics Data Repository investigated implementation barriers to molecular testing in advanced NSCLC and identified clinical practice gaps at the stage of biopsy referral, biospecimen collection, biospecimen evaluation, biomarker test ordering, biomarker test performance, test result reporting, and treatment decisions. Specifically, the authors highlight that 136 out of 934 (14.5%) late stage NSCLC patients in the study are “lost” during the patient progression from diagnosis to treatment selection due to poor biospecimen collection [30].

Three potential solutions may be of interest for future implementation studies based on the projected low implementation cost. First, improving the biopsy request with the addition of a “checkbox” in the electronic health record to indicate the need for molecular testing would facilitate coordination between radiologists, pathologists, and referring providers. Knowing that molecular testing is the goal of the biopsy would affect the biopsy procedure and specimen processing in that radiologists would be prompted to collect more tissue and pathologists could optimize tissue processing. Second, as already studied by Lim et al. educating referring clinicians on the importance of molecular testing may increase testing rates [28]. Specialty-specific education programs reviewing data and guidelines on molecular testing previously achieved a 12% increase in molecular testing among diagnostic specialists in Ontario [10]. Third, creating feedback loops that present biopsy results (including true negatives versus insufficient samples) to radiologists and pathologists would help inform data-driven discussions of biopsy results and patient outcomes at multidisciplinary tumor boards.

Potential solutions in our study were geared towards crafting a framework that promotes successful molecular testing, including strategies to increase the yield of the biopsy procedure itself, which could be disseminated via workshops at specialty society meetings in addition to the literature. Biopsies should be performed using coaxial technique and target the periphery of large tumors to avoid central necrosis [31]. Review of pre-procedural imaging, such as fluorodeoxyglucose-PET and contrast-enhanced CT, facilitates the identification of necrotic areas [31]. The shorter procedure time afforded by real-time CT, often referred to as “CT fluoroscopy,” creates time for ROSE and the collection of additional samples [32]. A minimum of at least four tissue cores was recommended by the panel, exceeding the minimum of three recommended by the CAP guideline since research has demonstrated that sensitivity for histopathologic diagnosis and molecular testing increases significantly between the second and fourth samples [24, 33–35]. ROSE remains best practice according to the literature [36], despite the limitations noted by the panel. FNA was generally not performed but may have a role in facilitating ROSE [37]. However, touch preparations of tissue cores offer an alternative to ROSE based on fine needle aspirates, and use of touch preparations is supported by the CAP guidelines on handling lung needle biopsy specimens [24]. Limiting the need for re-biopsy was a valid concern among radiologists. However, it should be noted that repeat lung needle biopsies are generally safe and may be required to realize the potential offered by novel systemic therapies [19, 20, 38].

Potential solutions to optimize specimen analysis relate to tissue allocation techniques and testing methods. Herbst et al. advocate using minimal tissue for histology to conserve tissue for molecular testing and developing a histology protocol that allocates sections up front for molecular and immunohistochemistry analyses [39]. The National Cancer Institute Molecular Analysis for Therapy Choice trial similarly recommends splitting each core into two specimens, one for diagnosis and one for ancillary studies such as molecular testing [40]. During ROSE, pathologists and cytotechnologists should use specific terminology to distinguish between “diagnostic adequacy” versus “ancillary testing adequacy” [33]. Optical coherence tomography may, one day, be used to perioperatively quantify viable tumor cells versus scar in tissue cores [41]. NGS panels offer many advantages over sequential single-gene or “hotspot” assays (e.g., tests for only *EGFR*, *ALK*, and *ROS1*) [8]. Multiple “hotspot” assays can quickly exhaust available tissue and lead to false negatives due to tissue depletion [21, 29, 39, 42–44]. A recent study of advanced NSCLC found that NGS panels had a 39% lower rate of unsuccessful genotyping, resulting in 30% fewer missed treatment opportunities compared to non-NGS assays [18]. Although NGS may take several weeks to process, rapid *EGFR* testing workflows have been reported and allow treatment with *EGFR*-directed therapies within days, all without compromising NGS workflows [23]. Archiving tissue for future molecular testing was proposed as another potential solution; an important limitation to this approach is that tissues may lose antigenicity over time [2, 45], and characteristics of advanced tumors may change after treatment [46].

The limitations of this study should be interpreted within the context of its design and the absence of oncologists and pulmonologists. The questionnaires were designed by professional market researchers; this may have led to biases in the questionnaires influencing participant responses. However, by outsourcing the research, design, and execution of the study, inherent biases held by participants were avoided. Participation was voluntary; thus, the panel was susceptible to self-selection bias. The intentional selection of physicians who had helped develop the current CAP guidelines may have skewed responses, but the inclusion of academic and community practices provided a diverse perspective. Although the sample size was limited, participants from across the United States ensured geographic diversity.

Increased molecular testing is required to harness the benefits of novel targeted therapies and immunotherapy for patients with advanced NSCLC. Barriers and potential solutions to obtaining percutaneous lung needle biopsy specimens for successful molecular testing in patients with advanced NSCLC relate to the biopsy request, biopsy procedure, specimen analysis, and communication. As the number of therapies continues to grow, the need to overcome these barriers to performing molecular testing requires a coordinated effort among radiologists, pathologists, and referring clinicians. Implementation studies should aim to investigate the efficacy of the proposed potential solutions.

## Data Availability

Anonymized transcripts of participant interviews from EDGE Research is available upon request.

## Abbreviations

CAP: College of American Pathologists
CT: Computed-tomography
NCCN: National Comprehensive Cancer Network
FNA: Fine needle aspiration
NSCLC: Non-small cell lung cancer
PET: Positron emission tomography
ROSE: Rapid on-site evaluation
